# Thirdhand Cigarette Smoke: Factors Affecting Exposure and Remediation

**DOI:** 10.1371/journal.pone.0108258

**Published:** 2014-10-06

**Authors:** Vasundhra Bahl, Peyton Jacob, Christopher Havel, Suzaynn F. Schick, Prue Talbot

**Affiliations:** 1 Department of Cell Biology and Neuroscience, University of California Riverside, Riverside, California, United States of America; 2 Environmental Toxicology Graduate Program, University of California Riverside, Riverside, California, United States of America; 3 Department of Clinical Pharmacology, University of California San Francisco, San Francisco, California, United States of America; 4 Department of Medicine, Division of Occupational and Environmental Medicine, University of California San Francisco, San Francisco, California, United States of America; Rajiv Gandhi Centre for Biotechnology, India

## Abstract

Thirdhand smoke (THS) refers to components of secondhand smoke that stick to indoor surfaces and persist in the environment. Little is known about exposure levels and possible remediation measures to reduce potential exposure in contaminated areas. This study deals with the effect of aging on THS components and evaluates possible exposure levels and remediation measures. We investigated the concentration of nicotine, five nicotine related alkaloids, and three tobacco specific nitrosamines (TSNAs) in smoke exposed fabrics. Two different extraction methods were used. Cotton terry cloth and polyester fleece were exposed to smoke in controlled laboratory conditions and aged before extraction. Liquid chromatography-tandem mass spectrometry was used for chemical analysis. Fabrics aged for 19 months after smoke exposure retained significant amounts of THS chemicals. During aqueous extraction, cotton cloth released about 41 times as much nicotine and about 78 times the amount of tobacco specific nitrosamines (TSNAs) as polyester after one hour of aqueous extraction. Concentrations of nicotine and TSNAs in extracts of terry cloth exposed to smoke were used to estimate infant/toddler oral exposure and adult dermal exposure to THS. Nicotine exposure from THS residue can be 6.8 times higher in toddlers and 24 times higher in adults and TSNA exposure can be 16 times higher in toddlers and 56 times higher in adults than what would be inhaled by a passive smoker. In addition to providing exposure estimates, our data could be useful in developing remediation strategies and in framing public health policies for indoor environments with THS.

## Introduction

Thirdhand smoke (THS) consists of residual tobacco smoke that sorbs to indoor surfaces and remains after the majority of the airborne components of the smoke have cleared. THS raises the concentration of nicotine and other smoke constituents in indoor environments occupied by smokers [1], [2]. During aging, the chemicals in THS can desorb back into the air or react to form new chemicals. For example, nicotine reacts with ambient nitrous acid (HONO) to form tobacco specific nitrosamines (TSNAs) [3], [4]. Exposure to THS and remediation of buildings and vehicles contaminated with THS have received little attention in the past and are important, especially in light of recent health-related studies that indicate the potentially hazardous nature of THS [5]–[8]. Because THS affects individuals with unknown or unwanted exposure, it is an issue with public health implications [2].

The negative health effects of active smoking and secondhand smoke exposure have been analyzed *in vitro*, in animals, and studies of human volunteers and populations [9]–[13]. Active smoking and secondhand smoke exposure adversely affect health across all age groups [9], [14], [15]. In contrast, little is known about the level of human exposure to THS and the resulting health effects. THS exposure can occur through the skin, by ingestion, and by inhalation. Infants and small children could be at greater risk than adults because their skin is thinner, their surface to volume ratio is higher, and because they spend more time in contact with THS-contaminated surfaces and where they can mouth THS-contaminated objects. If ingested, the fraction of THS that is soluble in saliva and digestive fluids will be available for intake (passage into the body but not across absorptive barriers) [16]. The extent of intake will depend on the concentration of THS chemicals, the fraction of THS that is in the air and on surfaces, and their solubility in saliva or sweat. The concentration of THS chemicals will vary with the number of cigarettes smoked in the room, the air exchange rate, and the time elapsed since smoking. Therefore, when evaluating exposure, it is important to consider that THS is dynamic and that aging can change the composition of THS over time.

Remediation, which is the removal of THS residue from surfaces in indoor environments or the safe containment of THS, is another important aspect of THS contamination that needs study [2], [17]. Methods of remediation will depend upon the level of contamination as well as the type of material. The materials commonly found indoors, such as natural and synthetic fibers, carpets, paper and wall board, each differ in their capacities to adsorb, absorb, bind, and release THS chemicals (unpublished data).

As a first step to understanding the persistence of THS in indoor environments, potential human exposures, and options for remediation, we repeatedly exposed cotton and polyester fabrics to cigarette smoke in an experimental chamber, stopped exposure and aged the fabrics for up to 19 months, then measured the concentrations of nicotine, nicotine-related alkaloids and tobacco-specific nitrosamines in extracts of fabrics. We tested chemical concentrations in both organic and aqueous solvent extracts, and then used the resulting data to model exposures that toddlers and adults could receive in environments containing THS.

## Methods

### Exposure of fabric to cigarette smoke

100% cotton terry cloth, and 100% polyester fleece were purchased at retail and washed three times in a domestic washing machine using an unscented, enzyme-free laundry detergent (Country Save powdered laundry detergent, Arlington, WA) in hot water with two rinses/cycle, and washing again with no detergent. These fabrics were chosen as they are commonly used in household products and in clothing. After line drying, fabrics were hung in a 6 m^3^ stainless steel chamber at UCSF and exposed to cigarette smoke as described previously in detail [18]. Briefly, smoke generated by an automatic smoking machine (Model TE-10z, Teague Enterprises, Woodland, California, USA) was diluted into conditioned, filtered air, and conducted through a 6 m^3^ stainless steel smoke aging chamber. The aging chamber contained three vertically staggered baffles and two internal fans to promote mixing. The cloth samples were hung on the baffles. Marlboro Red cigarettes were smoked according to ISO protocol 3308: 2012. Marlboro Red was chosen as it is the best-selling cigarette in the United States and is popular worldwide. Particle concentration at the outlet of the smoke aging chamber was measured using a laser photometer calibrated gravimetrically (Dusttrak II, model 8530, TSI Inc., Shoreview MI). Aerosol flow rates through the chamber were measured using an air velocity transmitter (model 641-b Dwyer Instruments, Michigan City IN). After smoking, air flow was turned off, and the chamber was closed while it still contained detectable levels of smoke. Smoke was generated 0–8 times/month according to the needs of ongoing clinical research between experiments. The time that the cloth sample was in the chamber, the number of hours of smoke, the average particle concentration for each experiment and the air velocity through the chamber were logged. The total particle mass that the fabric was exposed to was calculated as 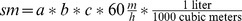 where a = air velocity in liters/minute, b = hours of smoke, and c = average smoke particle concentration.

A sheet of cotton terry cloth and a sheet of polyester fleece were exposed to smoke. The terry cloth was exposed to smoke containing 1329 mgs of particles for 114 hours over 1 year. The polyester fleece was exposed to 1846 mgs of smoke particles for 257 hours over 10 months. Both fabrics were folded and stored in separate polyethylene bags at room temperature in the dark. The terry cloth was stored 8 months and the polyester 1 month prior to shipment. Samples were wrapped in aluminum foil, placed in polyethylene bags and shipped at ambient temperature, overnight, to the Talbot Laboratory at UCR. Upon receipt, samples were transferred to amber glass bottles and stored at room temperature (RT) in the dark.

### Organic solvent extractions

Samples of fabric were incubated at RT overnight in 50% MeOH/1% HCl then vortexed for 3–5 minutes at RT. Solvent was removed by squeezing the fabric in the vial with a spatula, fibers and dust were removed by centrifugation, and the extract was analyzed as described below.

### Aqueous extractions

Aqueous extracts of THS were prepared in Dulbecco's Modified Eagle Medium (DMEM). Terry cloth and polyester fleece were weighed and cut into very small pieces using scissors. Either 0.05 or 0.125 g of fabric/ml were extracted in DMEM in 15 ml conical tubes on a rotating shaker. The medium was recovered by placing the fabric in a syringe and centrifuging at 4,000 g for 5 minutes The recovered medium was passed through a 0.22 µm filter, aliquoted into 1.5 ml vials, and stored at −80°C.

To examine the effect of repeated aqueous extraction on chemical yield, three samples of terry cloth and two samples of polyester were extracted five times, serially at RT with media being replaced every hour for 5 hours. To determine the effect of time and temperature, the terry cloth was extracted under four conditions: RT for 1 hour; RT for 2 hours; 4°C for 1 hour; and 4°C for 2 hours. For extraction at 4°C, tubes were placed in a beaker of ice on a rocker shaker. To examine the effect of aging, extraction was done after storing the terry cloth for 11, 16 and 19 months and the polyester for 11 and 19 months in amber glass jars at RT.

### Chemical Analysis of THS extracts

1 mL extracts of THS were shipped to UCSF on dry ice where they were analyzed using liquid chromatography-tandem mass spectrometry (LC-MS/MS) [19], [20]. The method was modified to include NNA in the analysis, by treating the extract with pentafluorophenylhydrazine (PFPH) to convert NNA to the pentafluorophenylhydrazone derivative which enhances sensitivity of detection [21].

### LC-MS/MS

The samples were analyzed on a Thermo Scientific Vantage LC-MS/MS with an Accela UPLC system using a 3×150 mm 2.6 micron Phenomenex Kinetex PFP column as detailed in [20].

### Limits of quantification

The limits of quantitation for each of the chemicals analyzed are as follows: nicotine: 1.02 ng/ml; myosmine: 0.305 ng/ml; 2,3′-bipyridine: 0.914 ng/ml; cotinine: 0.914 ng/ml; N-formylnornicotine: 0.305 ng/ml; nicotelline: 0.030 ng/ml; NNN: 0.030 ng/ml; NNK; 0.0130 ng/ml; NNA 0.010 ng/ml.

### Statistical analyses

The concentrations of chemicals in aqueous extracts were converted to grams/gram of fabric. Averages of four samples in each group were then calculated using Microsoft Excel. ANOVA (one way analysis of variance) was performed using GraphPad Prism to determine if the chemical concentrations in extracts made under different conditions varied significantly. ANOVA was also used to analyze extracts made from terry cloth after 11, 16 and 19 months of aging. Groups differing significantly (p<0.05) from the 11 month samples were identified using Dunnett's posthoc test. Data were checked to determine if they satisfied the assumptions of ANOVA (normal distribution and homogeneity of variances). T-tests were used to determine if the chemical concentrations in aqueous extracts were different from those in methanol/HCL extracts.

## Results

### Fabrics used for extraction

**Figure 1:**
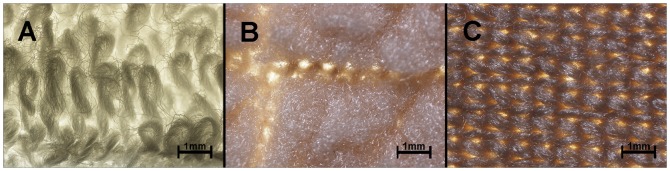
Micrographs of fabrics used for THS extraction. (A) Terry cloth is a loosely knit fabric made of loops of cotton which increase its surface area tremendously and contribute to the absorption of THS. (B) Polyester is a more tightly knit fabric with one fuzzy surface and (**C**) one compact tightly woven surface.

THS was extracted from 100% cotton terry cloth and 100% polyester fleece. Terry cloth is a loosely knit natural fabric with many thin fibers that provide a large surface area for absorption of chemicals. One surface of polyester has numerous short highly packed fibers while the other is comprised of a large tightly woven mesh of fibers (Fig. 1).

### Aqueous and methanol∶HCl solvents extracted THS chemicals from cotton fabric

**Figure 2:**
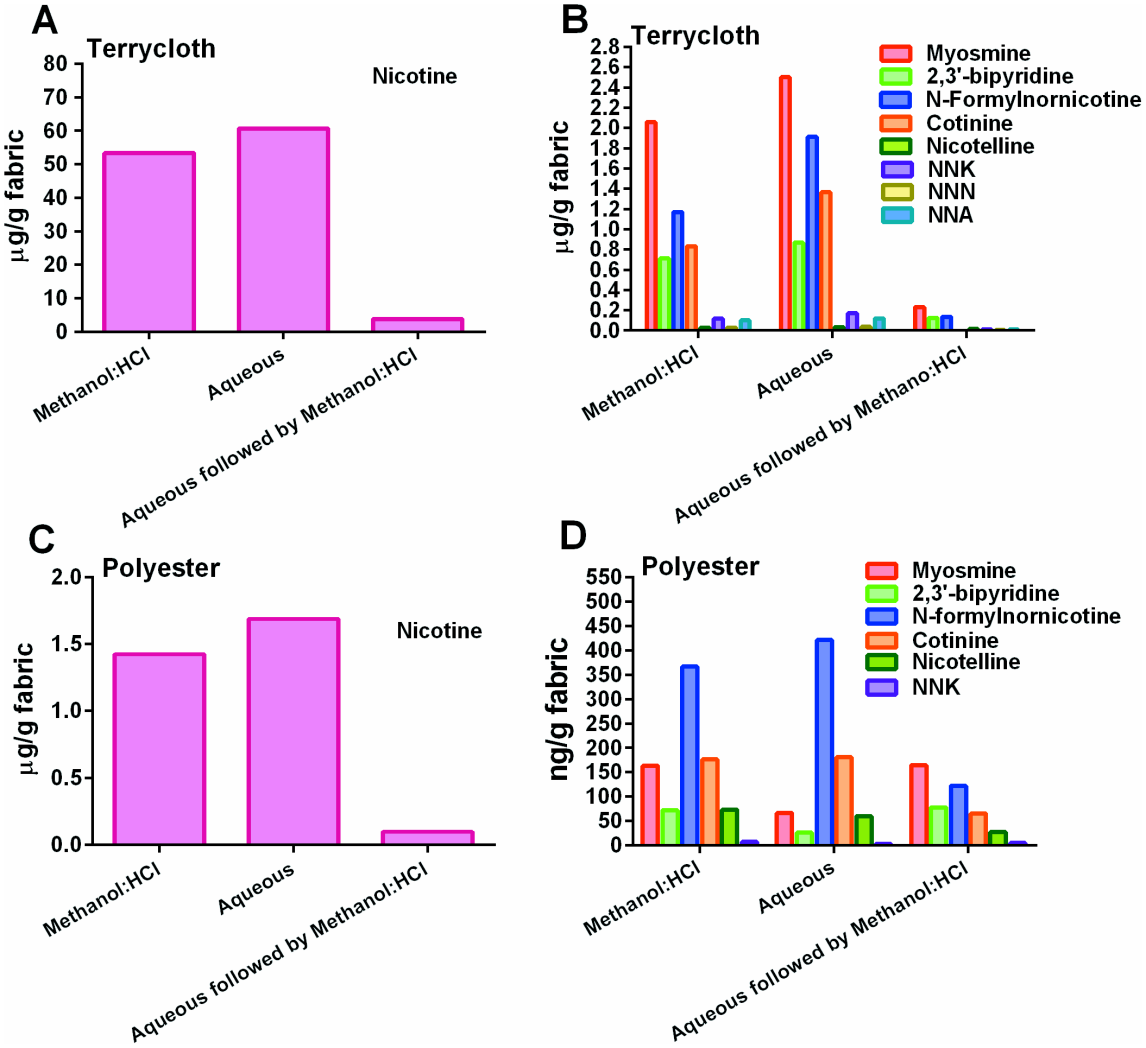
Comparison of chemical concentrations in aqueous and methanol∶HCl extracts of terry cloth and polyester exposed to THS. Terry cloth was extracted after 31 months of aging and polyester was extracted after 19 months of aging. Results for aqueous extracts are an average of three experiments, and all other groups are averages of two experiments.

The concentrations of nicotine and related chemicals in the aqueous extracts of THS from cotton terry cloth after 31 months of aging were similar to those in methanol∶HCl extracts (Figs. 2A, B). Negligible amounts of nicotine and related chemicals were recovered when aqueous extraction was followed by methanol∶HCl extraction. Nicotine (50–60 µg/gram of fabric) was the most abundant of the chemicals analyzed. Myosmine, bipyridine, formylnornicotine and cotinine were present in 1–2 µg/gm of fabric quantities, while the TSNAs and nicotelline were the least abundant (nanogram/gram of fabric) of the chemicals analyzed in THS extracts from terry cloth.

### Extraction of polyester fabric yielded lower concentrations of THS chemicals

The concentrations of all chemicals tested were lower in extracts of polyester fleece than in extracts of cotton terry cloth (Fig. 2C, D). As an example, in aqueous extracts approximately 40 times less nicotine was extracted from polyester than from terry cloth. For polyester fleece, methanol∶HCl and aqueous extracts had similar concentrations of nicotine and other chemicals. However, when aqueous extraction was followed by methanol∶HCl extraction, higher concentrations of myosmine and 2,3′-bipyridine were obtained than with aqueous extractions alone. All other chemicals were retrieved at lower concentrations in the methanol∶HCl extract that followed the aqueous extraction. This suggests two possibilities: that polyester binds less nicotine, nicotine- related alkaloids and TSNAs than cotton or that these compounds are harder to extract from polyester than from cotton.

### Serial aqueous extractions from terry cloth and polyester

**Figure 3:**
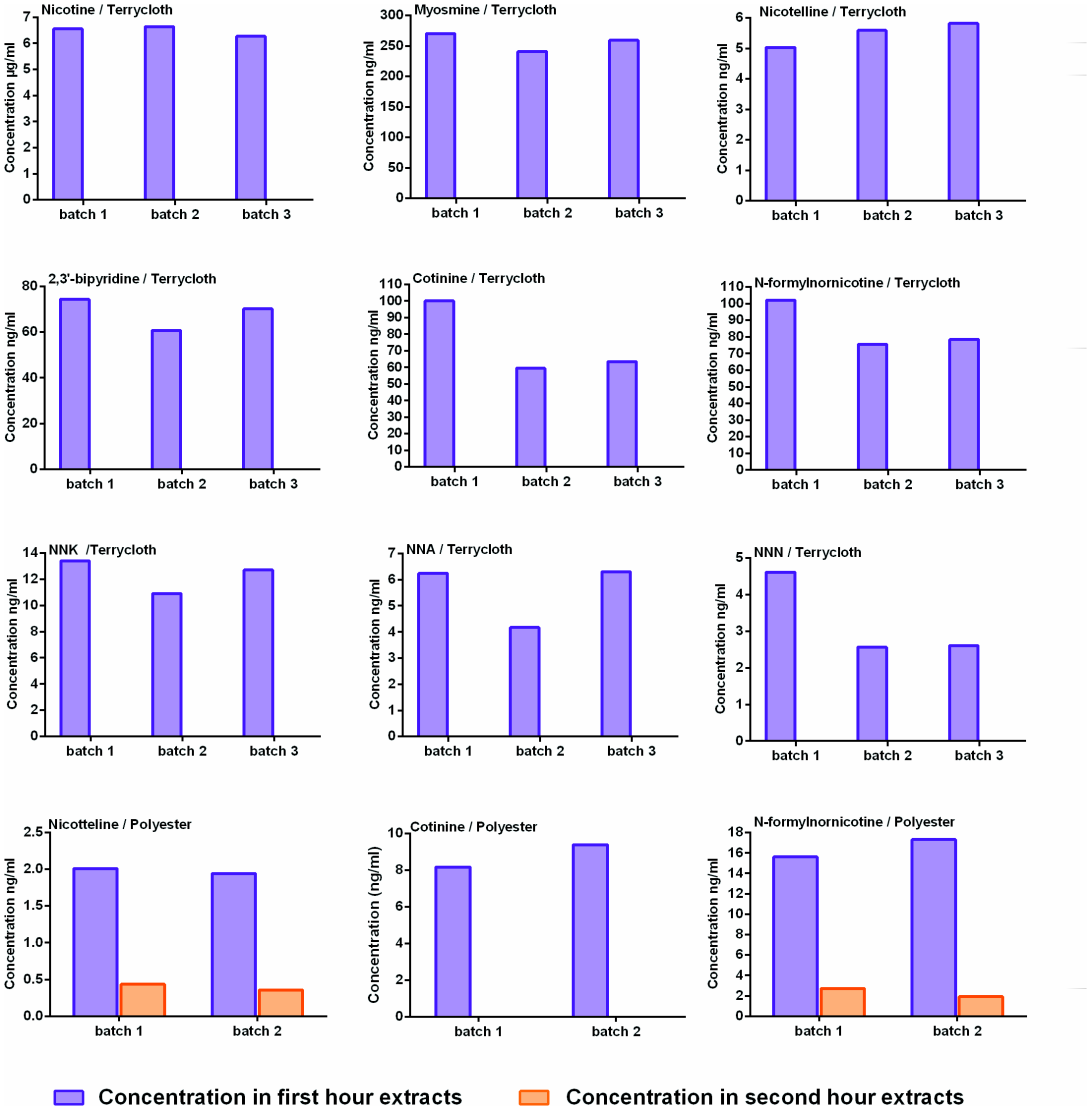
Iterative aqueous extractions from terry cloth and polyester. Extractions were done for a total 5 hours with extraction medium being replaced every hour. After every hour, extracts were analyzed for nicotine and its derivatives. Graphs represent chemical concentrations in three different batches of extracts. No chemicals were found in extracts after 1 hour for terry cloth and after 2 hours for polyester.

To determine if all nine chemicals were removed from terry cloth and polyester during 1 hour of aqueous extraction, the same fabric samples were extracted five times. Each extraction lasted one hour (Fig. 3). All of the chemicals extractable by water were successfully removed from cotton terry cloth during the first hour of extraction. Concentrations of some chemicals (e.g., nicotine, myosmine and nicotelline) were very similar from batch to batch, while others, such as cotinine, NNA, and NNN, varied somewhat in concentration among batches. For polyester, cotinine was found only in the first hour extracts. Nicotine and N-formylnornicotine were found in the first and the second hour extracts.

### One hour of aqueous extraction at RT removes THS chemicals from cotton terry cloth

**Figure 4:**
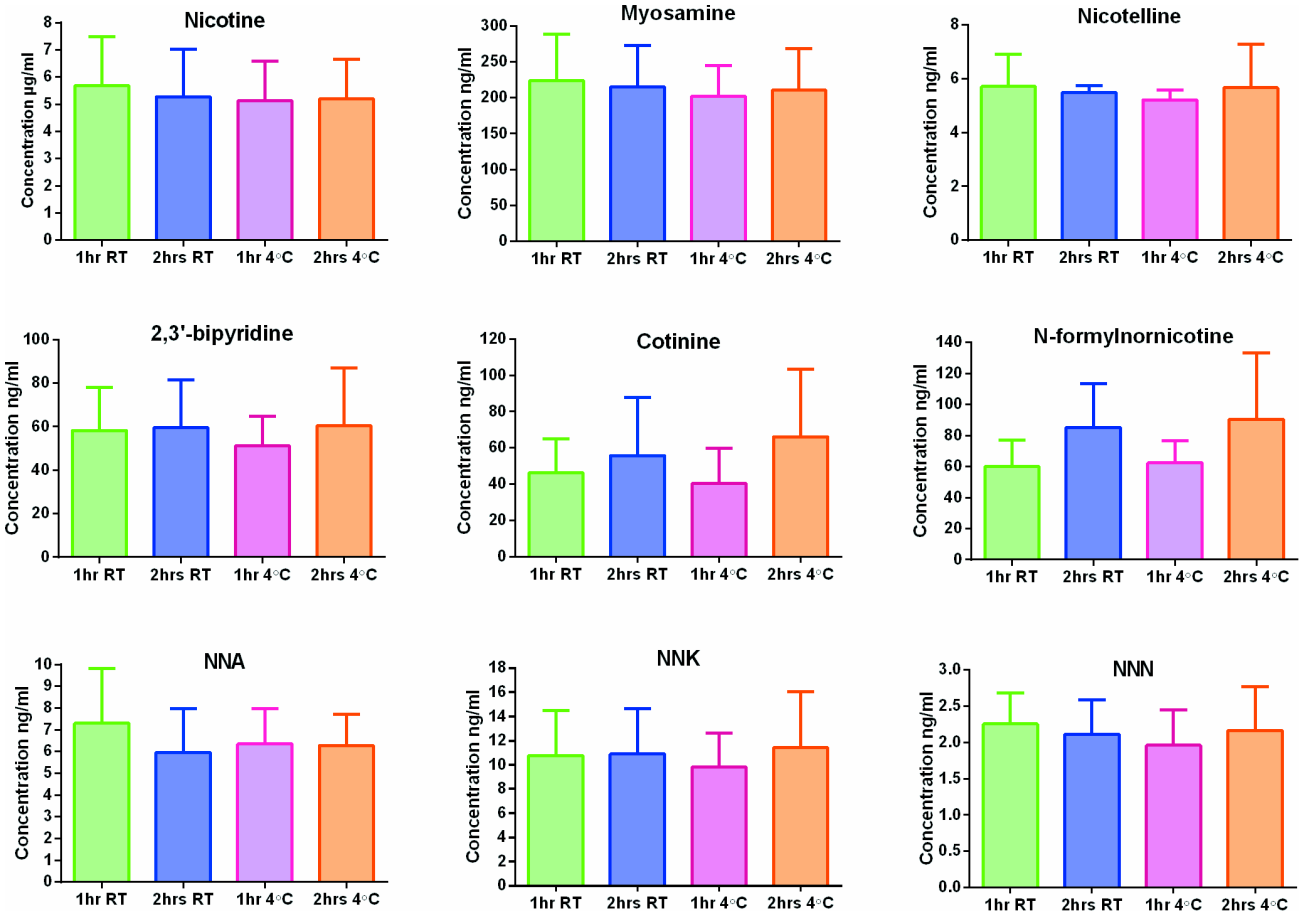
Concentration of chemicals in aqueous extracts of THS from terry cloth when temperature and time of extraction were varied. Extracts were made at RT and at 4°C for 1 and 2 hours. Each bar is the mean ± standard deviation of three experiments. Chemical concentrations did not vary significantly with temperature or time of extraction when tested by ANOVA.

The effects of temperature and time on the concentration of chemicals recovered by aqueous extraction was tested (Fig. 4). Extracts were made at RT and at 4°C for 1 or 2 hours. Chemical concentrations appeared to be similar for each extract. When tested by ANOVA, no significant differences in chemical concentrations were found between extraction conditions. Data for each chemical were therefore combined in Table 1, which also includes the combined data for polyester. These data confirm that 1 hour at RT is sufficient time to achieve the maximum yield of each chemical from cotton terry cloth using aqueous medium and that changing the time or temperature does not improve extraction efficiency. All chemicals were more abundant in extracts of terry cloth than in polyester, and NNN and NNA were not detected in the extracts of polyester.

**Table 1:** Chemicals identified in aqueous THS extracts.

Chemical	Terrycloth THS aqueous extract			Polyester THS aqueous extract	
	May 2012	Oct 2012	Jan 2013	Jan 2013	Sept 2013
**Nicotine µg/g**	105.8±25.5	112.92±8.59	69.6±30.4	0.557±0.82	1.689
**Cotinine µg/g**	0.899±0.13	1.04±0.14	0.446±0.04	0.269±0.06	0.18
**N-formylnor-nicotine µg/g**	3.9±0.72	1.138±0.13	1.047±0.27	0.427±0.09	0.420
**Myosmine µg/g**	4.844±0.31	4.518±0.25	3.010±0.12	X	0.066
**2,3′-bipyridine µg/g**	1.242±0.08	1.196±0.03	0.681±0.06	X	0.026
**Nicotelline ng/g**	105.8±25.5	113.22±20.61	104.95±10.9	36.35±10.6	59.61
**NNA ng/g**	229.3±95.6	218.8±16.4	88.3±8.8	X	X
**NNK ng/g**	169.5±27.2	218.8±16.38	132.36±9.76	X	3.2
**NNN ng/g**	37.10±5.18	45.84±3.08	31.25±3.46	X	X

### Effect of aging on the concentrations of THS chemicals in extracts from terry cloth and polyester

**Figure 5:**
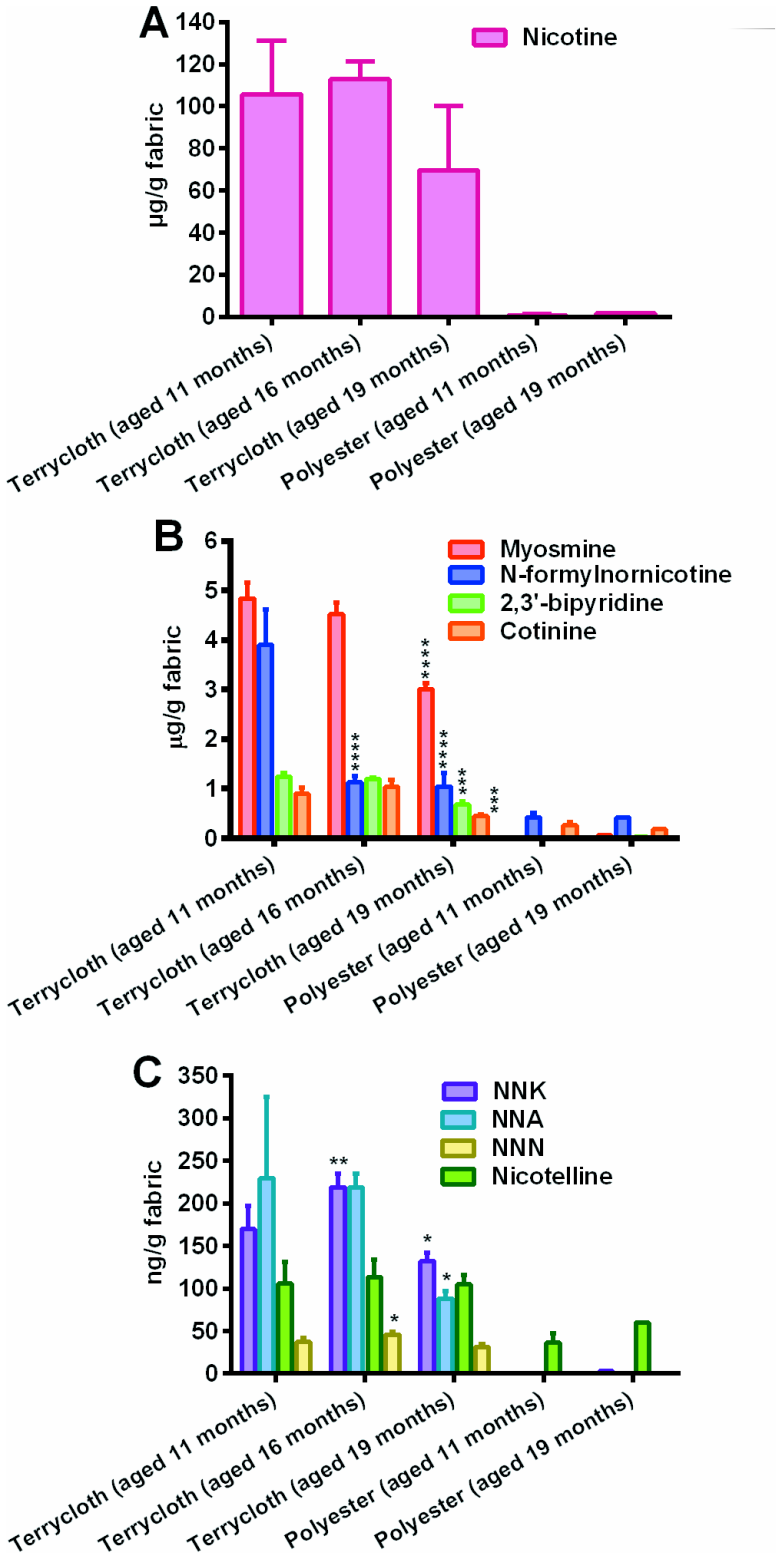
Effect of aging on the concentration of nicotine and its derivatives in aqueous extracts of terry cloth and polyester exposed to THS. Aqueous extracts of THS were made from terry cloth after 11, 16 and 19 months of aging and from polyester after 11 and 19 months of aging. (A) Nicotine and (B) Myosmine, N-formylnornicotine, bipyridine and cotinine were present in higher concentration than (C) nicotelline, NNK, NNA and NNN. Each bar is the average ± standard deviation of three extracts, except polyester (aged 19 months) which had only two experiments. ANOVA was used for testing significance followed by Dunnett's posthoc test in which comparisons were made to the samples aged for 11 months. **** p<0.0001; *** 0.0001<p<0.001; ** 0.001<p<0.01; * p<0.05.

In extracts of terry cloth, nicotine concentrations (105.8, 112.9, and 69.6 µg/gram of fabric) at 11, 16, and 19 months of aging (Fig. 5) were not significantly different when evaluated by ANOVA (p = 0.0595). Extracts of polyester made after 11 and 19 months of aging had very low amounts of nicotine (557 ng/g fabric and 168.8 ng/g fabric) in contrast to terry cloth that aged for similar times (Fig. 5A).

Myosmine, N-formylnornicotine, 2,3′-bipyridine, and cotinine were present in extracts of terry cloth at µg/gram of fabric concentrations (Fig. 5B). The concentrations of extractable myosmine (p<0.0001), 2,3′-bipyridine (p<0.0001) and cotinine (p = 0.0001) decreased significantly after 19 months of aging (January 2013). The concentration of N-formylnornicotine decreased significantly (p<0.0001) after 16 months of aging, but did not decrease further by 19 months. For this group of chemicals, the extract of polyester which aged 11 months contained only N-formylnornicotine and cotinine, and these were present in low concentrations compared to the corresponding terry cloth sample (11 months) (Fig. 3B). Extracts of polyester made after 19 months of aging had very low levels of myosmine, 2,3′-bypiridne, N-formylnornicotine and cotinine. All the chemicals in extracts of polyester were present at concentrations less than 1 µg/gram of fabric (Fig. 5B).

In extracts of terry cloth, concentrations of nicotelline, NNA, NNK and NNN were in the ng/gram of fabric range (Fig. 5C). Of these chemicals, only nicotelline did not decrease in concentration with aging, supporting its use as a tracer for tobacco smoke particulate matter [19]. The concentration of NNA decreased significantly by 19 months of aging (p = 0.0108). For both NNN and NNK, there was a slight but significant increase in concentration at 16 months of aging (p = 0.0020 and 0.0004 respectively), followed by a significant decrease in NNK (p = 0.0421) at 19 months.

In extracts of polyester made after 11 months of aging, nicotelline was detected in very small amounts, but the three TSNAs were absent. After 19 months of aging, very small amounts of NNK were also detected (Fig. 5C). Statistical analysis was not performed for extracts of polyester since the extract prepared after 19 months of aging had only two experiments.

## Discussion

While the concentrations of some extractable THS chemicals in cotton terry cloth and polyester fleece changed during aging, in general THS chemicals remained on these fabrics for over 1.5 years after the last exposure to smoke. Nicotine and its derivatives, including NNK, a known carcinogen, were rapidly extracted from cotton fabric in an aqueous medium that is similar in composition to saliva and sweat and has a physiological pH. This implies that an infant who mouths cloth that has been exposed to cigarette smoke will be exposed to significant amounts of cigarette smoke toxicants. There was a large difference in the quantity of chemicals extracted from cotton cloth and polyester cloth, showing that natural and synthetic fibers have different abilities to bind and release THS chemicals. These observations are important in understanding human exposure to THS, devising strategies for remediation of contaminated environments, and in developing regulatory policies for indoor use of tobacco products.

Changes in the concentration of an individual THS chemicals of on a surface depend on multiple processes including sorbtion, desorbtion and chemical reactions. Whether a chemical remains on a surface or rapidly desorbs and is removed by ventilation depends on its volatility and chemical properties. Whether a chemical reacts or remains intact depends on its chemical properties and the availability of other chemicals in the environment. With the exception of nicotelline, the chemicals we analyzed are semivolatile organic compounds, which means they will be present in both the gas phase and solid phase at normal indoor temperatures. For terry cloth, myosmine, 2′,3′;-bipyridine, N-formylnornicotine and cotinine decreased significantly during aging, possibly due to breakdown into other chemicals, volatilization, or conversion reactions with the ambient environment.

The increased concentrations of both NNN and NNK at 16 months of aging followed by a decrease at 19 months could be due to formation of fresh TSNAs from settled nicotine before reaching a threshold and starting to decrease due to further conversions. Although NNA concentrations did not change in our extracts, the formation of NNA may have occurred prior to our first extraction, after 11 months of aging. Also, NNA, being an aldehyde, is more reactive than NNN and NNK and could have combined with other chemicals during aging. For polyester, the concentrations of all chemicals in aqueous extracts were very low. The increase in the number of chemicals that were present in the polyester sample that aged 19 months vs. 11 months may be an artifact caused by analyzing chemicals close to their lower limit of quantification (0.01 ng/ml to 1 ng/ml for different chemicals).

**Figure 6:**
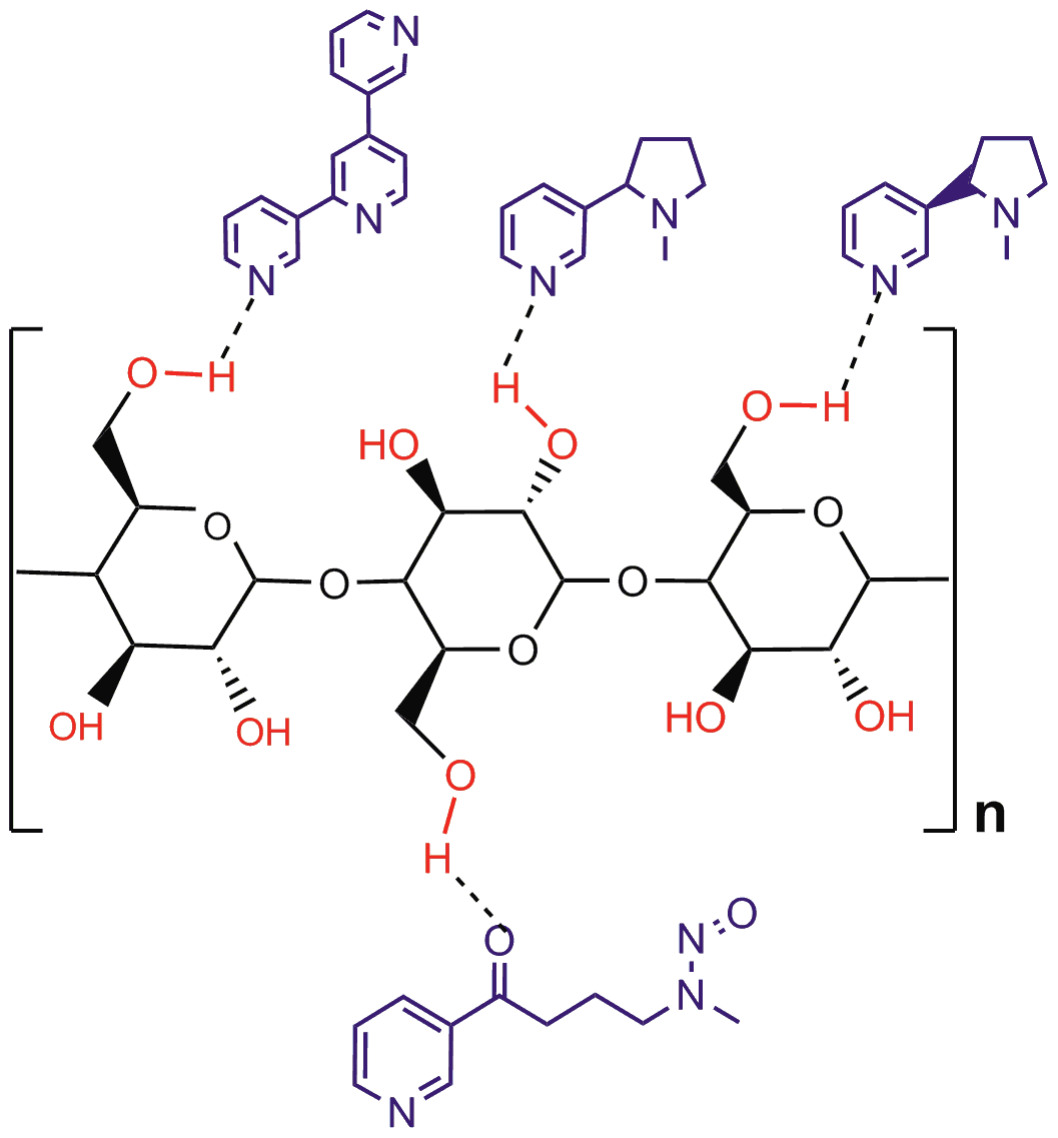
Chemical interactions of nicotine and its derivatives with terry cloth through hydrogen bonds. Terry cloth absorbs nicotine and related chemicals as these are polar in nature and can form hydrogen bonds with the free hydroxyl groups in terry cloth.

The difference in the concentrations of chemicals extracted from cotton terry cloth and polyester fleece may be due to their surface chemistry. Our data are in agreement with prior studies showing that polar substances like nicotine and dyes do not bind well to polyester [22], [23]. Cotton, which is made of cellulose, has three free hydroxyl groups/glucose monomer that can form hydrogen bonds with the polar groups on nicotine and its derivatives (Fig. 6) [22], [24], [25]. In contrast, polyester which is a polymer of terepthalic acid and ethylene glycol, is highly oleophilic [26], and its hydrophobicity tends to repel polar compounds. However, it is possible that these THS compounds adsorb more strongly to polyester than to cotton and the DMEM or methanol∶HCl extractions we used are not rigorous enough to fully extract them from polyester. THS contains thousands more chemicals than we analyzed, including many non-polar, non-water soluble chemicals. The interactions of other classes of chemicals with indoor surface materials will need to be characterized in future studies.

Our data clearly show that fabrics found in indoor environments act as reservoirs for THS smoke chemicals. Although the samples used in this study received relatively light exposure to cigarette smoke, significant amounts of nicotine and related chemicals were extractable from cotton cloth 19 months after smoke exposure had stopped. In studies where smoke is released into a large chamber and allowed to mix with the air, age and interact with surfaces before particle concentrations are measured, the mass of particles emitted by a single cigarette ranges from 7–22 mg, with averages between 8 and 14 mg [27]–[30]. Using an average emission factor of 10 mg per cigarette, the cotton cloth was exposed to the equivalent of 133 cigarettes and the polyester was exposed to the equivalent of 185 cigarettes. These exposures translate to 7–9 days of exposure in a room where 20 cigarettes are smoked per day or 27–37 days of exposure in a room where 5 cigarettes are smoked per day. Exposure in our study occurred in a steel chamber and therefore THS chemicals did not have an opportunity to be removed by ventilation, open doors or open windows. Therefore while our experiment was done under controlled laboratory conditions, it does not exactly duplicate a real world situation. However, the fact we used a low number of cigarettes (approximately 133 for terry cloth) supports the idea that in a real world situation the concentrations of the chemicals studied could be much higher than reported here. For example in a home where one individual smokes a pack a day for one year, the total number of cigarettes consumed would be 7,280 in contrast to approximately 133 used in our study. While the health effects of these chemicals in THS residue are not yet known, it may become desirable or even necessary in the future to remediate property with THS residue before it is rented or sold [2]. Our data demonstrate that nicotine and related compounds, including two carcinogens, can easily be removed from cotton fabrics by standard washing methods.

Since indoor surfaces act as reservoirs of THS, toddlers and infants could be exposed to THS chemicals by sucking on household fabrics, and all age groups could be exposed dermally by touching contaminated surfaces. To evaluate the exposure that could be received from cotton fabric containing THS residue, we examined a hypothetical scenario for dermal exposure to an adult. An adult wearing a 500 g cotton outfit containing THS residue from 20 cigarettes will be exposed to about 7,894 µg of nicotine/day and 32.7 µg of TSNAs/day, with a small fraction of this contributing to intake, assuming that the outfit would be washed frequently and could reasonably contain THS from 20 cigarettes before being washed.

A more accurate scenario can be developed for ingestion exposure to a toddler, where the intake will be roughly equal to the exposure. The main source of THS exposure to a toddler would be through mouthing fabrics used in toys, drapes and upholstery that are not frequently washed and have long-term accumulation of THS. For terry cloth containing THS from about 133 cigarettes (as used in this study), a 12 kg toddler mouthing and sucking 5 grams of cloth for 1 hour would be exposed to 529 µg of nicotine/day and 2.2 µg of TSNAs/day. Since the exposure and intake are equal, the toddler would receive 44 µg/kg body weight of nicotine and 0.183 µg/kg body weight of TSNAs per day. These intake values for the toddler would be less than those received by an active smoker but higher than respiratory exposure in passive smokers (6.8× higher for nicotine and 16× higher for TSNAs) (Table 2). While information on the effects of pre and postnatal nicotine exposure comes largely from animal models and women on nicotine replacement therapy, data consistently show links between nicotine exposure early in life and subsequent cognitive impairment, attention deficit disorders as well as obesity, hypertension, type-2 diabetes, respiratory dysfunction and impaired fertility [31]–[33]. Although the intake value for TSNAs is much less than doses known to cause tumors in rodent models [34], the above scenarios may underestimate exposure if significant levels of chemicals were lost during the first 11 months of aging or if THS accumulates from more than 133 cigarettes. TSNAs contribute to pancreatic cancer [35]. It will be interesting to determine in future studies if there is a correlation between TSNA exposure during infancy and the recent increase in pancreatic or other types of cancer in adults. Exposure of toddlers to nicotine and TSNAs in THS is therefore a matter of concern and may need regulation.

**Table 2:** Estimated nicotine and TSNAs exposure to a toddler.

Chemical	Inhalation Exposure Active Smoker	Inhalation Exposure Passive Smoker	Estimated Oral Exposure to THS
	Adult^a^	Toddler^b^	Toddler^c^
Nicotine	22,000 µg/day [36]	77.76 µg/day [37]	**529 µg/day**
TSNA	7.2 µg/day [38]	0.137 µg/day [39]	**2.2 µg/day**

a Based on smoking 1 pack/day.

b Based on respiration rate of 30 breaths/minute and tidal volume of 60 ml in a room with 30 µg/m^3^ nicotine and 53 ng/m^3^ of TSNA.

c Based on 1 hour of mouthing 5 grams of terry cloth exposed to 133 cigarettes and aged 19 months.

## Conclusions

Our data show that under controlled laboratory conditions fabrics exposed to cigarette smoke retain significant concentrations of THS chemicals long after smoking has ceased. Estimated exposure to and uptake of nicotine and TSNAs from residual THS are above what toddlers would receive by inhaling environmental tobacco smoke. These observations coupled with recent reports linking THS exposure to adverse health effects support the idea that THS residues on indoor surfaces are a public health concern. Since THS chemicals do not spontaneously disappear from indoor surfaces, it may be important to actively remove them to reduce risk from THS exposure. Our data show that nicotine, nicotine-related alkaloids and TSNAs could be readily removed from cotton fabrics by washing, which could become a simple remediation procedure.

This study focused on THS that had aged in fabrics that are often used in homes and clothing. Studies are in progress to determine the levels of chemicals in freshly exposed household fabrics, such as carpets, drapes and upholstery, as well as the actual intake and uptake levels of THS chemicals in humans and if these concentrations are high enough to produce harm.

## Acknowledgments

We thank Pura Tech for her help handling the samples.
